# Screening Practices for Infectious Diseases among Burmese Refugees in Australia

**DOI:** 10.3201/eid1511.090777

**Published:** 2009-11

**Authors:** Nadia J. Chaves, Katherine B. Gibney, Karin Leder, Daniel P. O’Brien, Caroline Marshall, Beverley-Ann Biggs

**Affiliations:** Royal Melbourne Hospital, Parkville, Victoria, Australia (N.J. Chaves, K.B. Gibney, K. Leder, D.P. O’Brien, C. Marshall, B.-A. Biggs); Monash University, Melbourne, Victoria, Australia (K. Leder); Médecins sans Frontières Holland, Amsterdam, the Netherlands (D.P. O’Brien); University of Melbourne, Parkville (C. Marshall, B.-A. Biggs); 1 These authors contributed equally to this article.

**Keywords:** Bacteria, viruses, parasites, Helicobacter pylori, hepatitis B, Myanmar, strongyloides, refugees, Australia, research

## Abstract

Increasing numbers of refugees from Burma (Myanmar) are resettling in Western countries. We performed a retrospective study of 156 Burmese refugees at an Australian teaching hospital. Of those tested, *Helicobacter pylori* infection affected 80%, latent tuberculosis 70%, vitamin D deficiency 37%, and strongyloidiasis 26%. Treating these diseases can prevent long-term illness.

## Abstract 2

*Helicobacter pylori* and *Strongyloides* spp. infections were the most common conditions found.

Burma (Myanmar) has been the most common country of origin for refugees who have recently resettled in the United States and Australia (*1**,**2*). Before resettling in Australia, most refugees undergo testing for HIV, have a chest radiograph to exclude active tuberculosis (TB), and may undergo other testing, depending on exposure risk. Many refugees also receive a health check and treatment for malaria and stool parasites within 72 hours of departure for Australia (*3**,**4*). Most refugees who resettle in Victoria, Australia, are screened by primary care doctors and then referred to specialist clinics as appropriate. In this study, we examined the effect of illness and the adequacy and completeness of health screening among Burmese refugees referred to the infectious diseases clinic of an Australian tertiary hospital during a 5-year period.

## Methods

We performed a retrospective cohort study of all Burmese refugees who attended the Victorian Infectious Diseases Service outpatient clinics at the Royal Melbourne Hospital, Australia, during January 1, 2004–December 31, 2008. Patients were identified through the hospital registration database, and medical, pathologic, radiologic, and pharmacologic records were reviewed. Screening tests audited included those suggested by the Australasian Society for Infectious Diseases refugee screening guidelines (*5*), along with vitamin D and hematologic studies. These latter tests included full blood count, mean corpuscular volume, and platelet count. Investigations were performed at the discretion of the treating doctor, and not all tests were performed for each patient. Time was calculated from time of arrival in Australia to first clinic attendance. The results of serologic tests and QuantiFERON-TB Gold tests (QFT-G; Cellestis Limited, Carnegie, Victoria, Australia), were interpreted according to the manufacturers’ recommendations.

Conditions were defined according to prespecified criteria as follows: schistosomiasis; strongyloidiasis; HIV and syphilis (positive serologic test results); hepatitis C virus (RNA detected by PCR); *Helicobacter pylori* (positive results for fecal antigen test, carbon-14 breath test, or serologic analysis); malaria (thick and thin blood films or immunochromatographic test result positive for *Plasmodium* species); chlamydia and gonorrhea (DNA detected by PCR in first-pass urine); active TB (microbiologic or histologic evidence of *Mycobacterium tuberculosis* infection or receiving treatment for active TB during the study period); latent TB infection (Mantoux test result >10 mm or positive QFT-G result and no clinical evidence of active disease); chronic hepatitis B virus (HBV; hepatitis B surface antigen detected); isolated core antibody against HBV (hepatitis B core antibody detected, hepatitis B surface antibody and hepatitis B surface antigen not detected); pathologic stool parasites (stool microscopy positive for a pathogenic species); vitamin D deficiency (serum 25[OH] vitamin D level <50 nmol/L); anemia (hemoglobin level <120g/L); and eosinophilia (eosinophil count >0.4 × 10^9^ cells/L). The Melbourne Health Human Research Ethics Committee approved this study as a quality assurance audit.

## Results

A total of 156 Burmese refugees were referred to the infectious diseases outpatient clinics at the Royal Melbourne Hospital during the study period. Table 1 summarizes the characteristics of these patients. Median age was 30 years (range 16–86 years); approximately half were male (51%) and of Karen ethnicity (48%). Most refugees were born in Burma (97%) and had spent time in a refugee camp (97%). The proportion of these patients who were screened according to the Australian refugee health guidelines is shown in the Figure. More than 90% of study patients were tested for 6 diseases (*Mycobacterium* TB, HIV, hepatitis B, hepatitis C, schistosomiasis, and *Strongyloides stercoralis* infection).

**Table 1:** Patient characteristics, Burmese refugees in Australia, 2004–2008*

Characteristic	Value
Age group, y, no. (%)	
<25	36 (23.1)
25–49	108 (69.2)
>50	12 (7.7)
Gender, no. (%)	
M	80 (51.3)
F	76 (48.7)
Country of birth, no. (%)	
Burma (Myanmar)	152 (97.4)
Thailand	4 (2.6)
Preferred language, no. (%), n = 155	
Burmese	23 (14.8)
Karen	74 (47.7)
Chin	55 (35.5)
English	2 (1.3)
Zotung	1 (0.6)
Transit through refugee camp, no. (%), n = 137	133 (97.1)
Country of refugee camp, no. (%), n = 135	
Thailand/Thailand-Burma border	71 (52.6)
Malaysia	55 (40.7)
Other	9 (6.7)
Referral by general practitioner, no. (%)	151 (96.8)
No. clinic visits per refugee, median (range)	5 (1–18)
No. months attended clinic, median (range)	8 (1–23)
No. months in Australia, median (range)	4 (<1–60)

*n = 156 unless otherwise specified.

**Figure:**
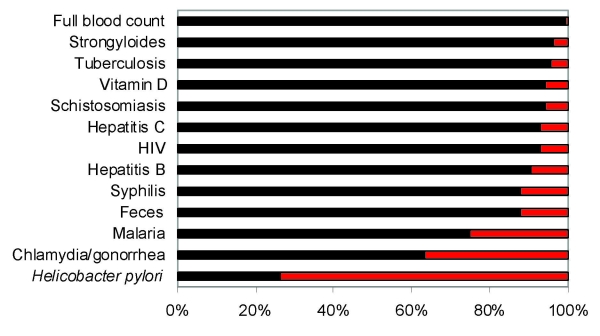
Proportion of 156 recently arrived Burmese refugees with documented screening tests for common health conditions, Australia, 2004–2008. Most of these tests are recommended by the Australasian Infectious Diseases Society guidelines (*5*). Tests for vitamin D levels are beyond the scope of these guidelines. Black, tested; red, not tested.

Table 2 shows the prevalence of selected medical conditions in this patient group. Chronic HBV infection was found in 14% of the group; isolated core antibody against HBV was found in 13%. Hepatitis B DNA was not detected in the serum of any patients with isolated core antibody against HBV. One person had HIV infection; this person had a chronic infection with HBV. *H*. *pylori* infection was identified in 80% of those tested (7 persons by carbon-14 breath test, 7 by fecal antigen test, and 19 by serologic analysis). No cases of multidrug-resistant TB were found.

**Table 2:** Proportion of patients with selected conditions compared with other studies of Burmese immigrants, retrospective cohort study, Australia, 2004–2008*

Condition	This study, no. positive/ no. tested (%), N = 156	Denburg study (*6*), % positive, N = 68	Minnesota Department of Health study (*7*), % positive, N = 159
*Helicobacter pylori* infection	33/41 (80.5)		
Latent TB	105/149 (70.5)	28	52
Vitamin D deficiency	55/147 (37.4)		
Eosinophilia	55/155 (35.5)	50	
*Strongyloides* infection (serology)	39/150 (26.0)	7.5	
Stool parasites (pathology)	33/137 (24.1)	32	18
Chronic HBV infection	20/141 (14.2)	13	9
Isolated core antibody against HBV	18/141 (12.8)		
Schistosomiasis (serology)	8/147 (5.4)		
HCV infection	4/145 (2.8)		
Active TB	3/149 (2.0)		
Syphilis	2/137 (1.5)	0	<1
Malaria	1/117 (0.9)		
HIV infection	1/145 (0.7)		
Chlamydia infection/gonorrhea	0/99 (0.0)		

*TB, tuberculosis; HBV, hepatitis B virus; HCV, hepatitis C virus.

Eosinophilia was documented in 35% of those tested, 47% of whom had strongyloidiasis, 4% schistosomiasis, and 24% a pathologic stool parasite that causes eosinophilia. Eosinophilia was not explained by these conditions in 33%.

## Discussion

In recent years, an increasing number of refugees from Burma have resettled in Australia, North America, and Europe. This study reports high rates of *H. pylori* infection (80%), latent TB infection (70%), vitamin D deficiency (37%), and strongyloidiasis (26%) in Burmese refugees attending the infectious diseases clinics of a Melbourne tertiary referral hospital.

A Canadian study of 68 Karen refugees, more than half of whom were <18 years of age, appears to be the only previously published study on the health status of Burmese refugees settled in a Western country (*6*). One unpublished study found on the Internet was conducted by the Minnesota Department of Health, which examined 159 Burmese migrants, but no demographic information was included (*7*). We have compared our screening results with those of these 2 studies in Table 2.

A high rate of parasitic intestinal infections has been documented in refugees from Burma in Thailand (*8**–**10*) and North America (*6**,**7*), and our findings are consistent with these studies. Parasitic intestinal infections were common in our study despite some refugees reporting that they had received predeparture drug therapy with albendazole. Therefore, we suggest that refugees migrating from Burma to Australia who underwent postarrival stool evaluation may not have received the predeparture antiparasitic, or if received, the treatment was ineffective. Moreover, infection with *S. stercoralis* was common in this study. This parasite is unlikely to be eradicated with only 1 dose of albendazole and is associated with chronic complications, including hyperinfection syndrome and death (*11*).

The rate of infection with *H. pylori* in this group was surprisingly high at 80% because the number of refugees tested was small and those tested were symptomatic. High rates of infection with *H. pylori* have been seen in other immigrant groups (*12**,**13*). This result reinforces the need to question refugees regarding dyspeptic symptoms and to test those with symptoms because of established links between *H. pylori* infection and iron deficiency, peptic ulcer disease, and gastric cancer (*14**,**15*).

National screening protocols for refugees were closely followed in this study for most infectious diseases. Lower compliance (<88%) with screening protocols was reported for malaria, chlamydia, and gonorrhea. However, these conditions were uncommon in this group. Ongoing education of health professionals who care for refugees is required to encourage more complete screening of refugees.

This study has a number of limitations. A relatively small number of patients were tested. Because this study was retrospective and screening was incomplete for some patients, certain diseases may be underestimated, or if testing was based on symptoms rather than true screening, diseases may be overestimated. Some conditions will be overrepresented because of referral bias and because certain tests (e.g., for *H. pylori*) were performed as diagnostic evaluation of symptomatic patients. This study highlights the difficulties in providing complete health screening for refugees, outlines the range of health problems among Burmese refugees referred to an adult tertiary hospital in Australia, and reinforces the high prevalence of treatable conditions in refugee communities.

## References

[1] Australian Government Department of Immigration and Citizenship. Fact sheet 60—Australia’s refugee and humanitarian program. 2008 [cited 2009 May 11]. Available from http://www.immi.gov.au/media/fact-sheets/60refugee.htm

[2] United States Department of Health and Human Services, Office of Refugee Resettlement. Fiscal year 2007 refugee arrivals. 2008 [cited 2009 March 30]. Available from http://www.acf.hhs.gov/programs/orr/data/fy2007RA.htm

[3] Australian Government Department of Immigration and Citizenship. Fact Sheet 67a—Pre-departure medical screening. 2008 [cited 2009 Feb 16]. Available from http://www.immi.gov.au/media/fact-sheets/67a_pdms.htm

[4] Foundation House—The Victorian Foundation for Survivors of Torture. Promoting refugee health—a guide for doctors and other health care providers caring for people from refugee backgrounds. 2007 [cited 2009 Mar 26]. Available from http://www.foundationhouse.org.au/resources/publications_and_resources.htm

[5] Australasian Society for Infectious Diseases. Diagnosis, management and prevention of infections in recently arrived refugees. Sydney (NSW, Australia): Dreamweaver Publishing Pty Ltd.; 2008 [cited 2009 Mar 26]. Available from http://www.asid.net.au/downloads/RefugeeGuidelines.pdf 10.5694/j.1326-5377.2009.tb02489.x 19374613

[6] Denburg A, Rashid M, Brophy J, Curtis T, Malloy P, Audley J, Initial health screening results for Karen refugees: a retrospective review. Can Commun Dis Rep. 2007;33:16–22. 18161207

[7] Chute S. Karen refugees from Burma. 2007 [cited 2009 Feb 16]. Available from http://www.health.state.mn.us/divs/idepc/refugee/metrotf/karenarrival.pdf

[8] Nuchprayoon S, Sandprasery V, Kaewzaithim S, Saksirisampant W. Screening for intestinal parasitic infections among Myanmar migrant workers in the Thai food industry: a high risk transmission. J Immigr Minor Health. 2009;11:115–21. 10.1007/s10903-008-9169-8 18815883

[9] Piangjai S, Sukontason K, Sukontason K. Intestinal parasitic infections in hill-tribe schoolchildren in Chiang Mai, Northern Thailand. Southeast Asian J Trop Med Public Health. 2003;34(Suppl 2):90–3. 19230577

[10] Saksirisampant W, Prownebon J, Kanmarnee M, Thaisom S, Yenthakam S, Nuchprayoon S. Prevalence of parasitism among students of the Karen Hill Tribe in Chame District, Chiang Mai Province, Thailand. J Med Assoc Thai. 2004;87(Suppl2):S278–83. 16083202

[11] Einsiedel L, Spelman D. *Strongyloides stercoralis*: risks posed to immigrant patients in an Australian tertiary referral centre. Intern Med J. 2006;36:632–7. 10.1111/j.1445-5994.2006.01172.x 16958639

[12] Cherian S, Forbes D, Sanfilippo F. The epidemiology of *H. pylori* infection in African refugee children. Med J Aust. 2008;189:438–44. 18928436 10.5694/j.1326-5377.2008.tb02116.x

[13] Gibney K, Mihrshahi S, Torresi J, Marshall C, Leder K, Biggs BA. The profile of health problems in African immigrants attending an infectious diseases unit in Melbourne, Australia. Am J Trop Med Hyg. 2009;80:805–11. 19407128

[14] Muhsen K, Cohen D. *Helicobacter pylori* infection and iron stores: a systematic review and meta-analysis. Helicobacter. 2008;13:323–40. 10.1111/j.1523-5378.2008.00617.x 19250507

[15] Suerbaum S, Michetti P. Medical progress: *Helicobacter pylori* infection. N Engl J Med. 2002;347:1175–86. 10.1056/NEJMra020542 12374879

